# Hypnotism

**Published:** 1894-08

**Authors:** Geo. H. Mead


### Hypnotism.

BY PROFESSOR GEO. H. MEAD.

Digest of paper read before the Thirty-eighth Annual Meeting of the Michigan Dental Association.

Treatment of disease by suggestion goes back even to prehistoric times. The means by which this suggestion was brought about was through the belief in the charms, ceremonials and rites practiced, but the principle was the same as in the most modern methods of hypnotic therapeutics.

Hypnotism can be approached from two sides—the psychical and the physiological. On the psychical we have to describe the state of consciousness of the person hypnotized, on the physiological the state of the physical organism during the condition.

The positive action of suggestion is perfectly normal. Every suggestion that we received, either through words spoken to us, or through signs given consciously or unconsciously, or through objects which suggest actions tends to be carried out, and if we follow out our obscure states of consciousness we will find these tendencies even when they are checked almost before we recognize them. Hypnotism consists in removing the checks and counter-suggestion by which we direct our actions. So far as we can describe such a condition there must be a great dimness of background and environment in the midst of which the single suggestion stands out with relative distinctness. The whole pathological condition in the hypnotic state consists in the rendering dim and indistinct of the background and environment of consciousness by means of which we judge of the value and propriety of actions and in which we find the counter-suggestions by which we check or direct any particular suggestion.

On the physiological side we must suppose a change in the co-ordination of the central nervous system, such that one line of central reflex is not connected with the other paths, which would, under ordinary circumstances, check or modify or direct its expression. Further than such a general account we are not as yet able to go in any physiological statement of the hypnotic state.

On the therapeutical side we are justified in using hypnotism for all those diseases in which suggestion has worked already in producing pathological conditions, and also in such functional diseases as allow of regulation through suggestion acting by means of the higher nervous centers. But especially is hypnotism allowable in cases where, as in dentistry, there is need of a local obtundent or anæsthetic.

DISCUSSION.

DR. PARKER, Grand Rapids, Mich.: I would like to cite a case of hypnotism. The patient was a person of more than ordinary ability, clear sighted, not easily deceived. She had made a study of this subject to some extent. She came to me for an operation. As her teeth were extremely sensitive I determined to hypnotize her. I told her this. I told her I wanted her to go to sleep, not entirely asleep, but just far enough to dream. After two or three minutes I said to her "Here is a book. I want you to take it and go down to M——. There is a hammock down there and I want you to have a good time." I then began filling the tooth. I worked for about two hours—it was a large gold filling. After finishing my work, I woke her up by snapping my fingers. She awoke with a start. "Oh, doctor, I have had such a nice time. I have been down to M——. I had a hammock there and such a nice book to read." I asked her what the book was about. "I do not recall any particular part of it, but it was very interesting." She was much surprised when I told her what I had been doing, and of course was very grateful.

Hypnotism is certainly a wonderful thing and a power for good, but it is also, in the hands of the unscrupulous, a power for ill.

DR. J. L. SWEETNAM, Manistee, Mich.: It is a question in my mind whether it is right or not for a person to use the hypnotic power. There is no question whatever about it being possible to put persons into a state of insensibility to pain by this means, but is it right for dentists to use this power? I became quite enthusiastic at one time about this subject and made use of it in my practice successfully. I could extract teeth, and live pulp without the least pain to the patient. But I found it reacted against me in the minds of my patrons, and fearing it would injure my practice I stopped it. What I want to find out, is, what position this practice is likely to take in dentistry. I do not wish to sacrifice my practice, nor do I wish to go on alone.

